# Severe plastic deformation of Al strips through equal multi and single channel angular pressing

**DOI:** 10.1038/s41598-025-88185-z

**Published:** 2025-02-15

**Authors:** Elshafey Ahmed Gadallah, Mohamed Ibrahim Abd El Aal

**Affiliations:** 1 Mechanical Department (Production), Faculty of Technology and Education, Suez University, Suez, 43221 Egypt; 2 Department of Mechanical Engineering, College of Engineering in Wadi Alddawaser, Prince Sattam Bin Abdulaziz University, Wadi Addawaser, 18734 Saudi Arabia

**Keywords:** ECMAP, ECAP, Al, 3D FEM, Load-displacement, Microhardness, Mechanical engineering, Mechanical properties

## Abstract

Equal channel multi-angular pressing (ECMAP) and equal channel angular pressing (ECAP) of Al-1050 strips were carried out. The ECMAP and ECAP load-displacement behavior, peak load, microhardness distribution maps, homogeneity, and average values were investigated. Finite element simulations (FEM) of the ECMAP and ECAP were performed using the DEFORM-3D program. The experimental and FEM load-displacement curves were close to each other, with deviations between the FEM and experimental peak loads of 2.3–9.5 and 0.9–3.1% in the ECMAP and ECAP. The ECAP experimental and FEM peak loads were lower by 149.4-163.4 and 127.9-151.8% than that of the ECMAP after one to three passes. FEM average effective plastic strain was obtained with high accuracy in the different plans. The ECAP sample’s deformation homogeneity was higher by 19-62.6% relative to that of ECMAP samples. The microhardness results verify the 3D FEM simulation one that ECAP samples have a higher deformation homogeneity. Moreover, the average microhardness values of ECAP samples were higher by 4.5–13.3% than the ECMAP samples in the different plans. ECAP processing of Al-1050 strips is recommended rather than using ECMAP to produce large samples with a higher degree of deformation homogeneity and microhardness.

## Introduction

Producing materials with nano and ultrafine grains (UFG) can be considered the motivation of different publications about severe plastic deformation (SPD) in the last 30 years. Equal channel angular pressing (ECAP) and high-pressure torsion (HPT) are used especially to process Al and Al alloys samples^1,2^. The ECAP is considered the most effective and popular SPD process due to the large size of the ECAPed samples^3–8^. The ECAP principles, processing routes, the deformation behavior during the ECAP, and the effect of the ECAP sample size were fully covered in different previous studies^3–8^. The principles of the ECAP process and the most important used processing routes and their effect on the deformation and shearing characteristics were fully investigated^3–8^ Interestingly, til now references^3–8^ can be considered the most important review studies about the SPD process, especially ECAP.

The equal channel multi-angular pressing (ECMAP) dies appeared due to the modification of ECAP dies^9^. ECMAP starts with the processing of bulk square samples^9^. The ECMAP of Al99.99% indicates a slight change in the microstructure and hardness relative to the ECAPed samples^9^.The previous works about the effect of using ECMAP extended through the comparison with using traditional ECAP dies and modified ECAP die that so-called ECMAP die by finite element simulations (FEM)^10,11^. Moreover, the effect of the ECAP and ECMAP processing using traditional dies on the applied load was also investigated^10,11^. The results indicate the effectiveness of route A over route C with the need for lower load in the case of the ECAP processing relative to that in the ECMAP one^10^.

The deep analysis of the results indicates an error in the declared conclusions^10^, that the strain imposed by both routes was approximately the same, with higher homogeneity in the case of route C^10^. The average values of the strain in the case of ECMAP using routes A and C must be corrected to 2.04 and 2.05. Moreover, the standard deviation of the strain values was 0.19 and 0.099 in routes A and C. Therefore, route C can provide a higher degree of homogeneity in the ECMAP with a lower peak load^10,11^. Interestingly, the ECAP under the same imposed strain produces more homogenized samples^12^. Furthermore, the peak load values of the ECAP were half of that noted in the case of the ECMAP.

Most of the previous works of the ECAP^1–8^ and ECMAP^9–11^ of Al are concerned with cylindrical and square cross-section shapes, including the tracing of microstructure evolution, mechanical properties enhancement, and FEM simulations. However, ECMAP processing of thin-thickness Al samples starts with limited previous works^12,13^. The effect of the ECMAP processing route on the load stroke curves, peak load, and strain distribution of Al5754 thin samples with a thickness of 3 mm was studied^12,13^. Route C can provide a higher deformation homogeneity and imposed strain than that of route A. The calculated average strain from the provided curves^13^, in the case of routes A and C of 0.49 and 0.52, obviously deviated from that obtained from the IWAHASHI Y Eq. (1)^14^ of 0.61. Average FEM strain values were deviated by 20 and 15% from the calculated strain^14^ in the case of routes A and C, respectively.

Although different Al and alloys were severely deformed in many previous works^1–6,9,12,13^. Al-1050 grade was selected as it is a popular grade of aluminum for general sheet metal work, available in the nearest shape of strips needed in the current study. Moreover, the moderate strength and high ductility of Al-1050 enable it to be formed up to multiple passes of ECAP and ECAMP, not only for one pass, as investigated before^9–13^. All the previous works concerned with the ECMAP of Al only through one pass^9–13^. Therefore, the effect of using the ECMAP for a higher number of passes is still needed, as no studies cover this area yet.

The FEM simulations in the current are concerned with 3D FEM simulations that didn’t use before in the FEM of ECMAP^10–13^, those performed by 2D FEM simulation^10–13^. Using 2D FEM provided strain distribution and deformation homogeneity in one dimension. However, 3D FEM in the current study can provide strain distribution and homogeneity in the three dimensions.

The selection between ECMAP and ECAP, as SPD techniques, is still confusing. Therefore, further investigations about the effectiveness of ECMAP processing of Al thin thickness samples (strips) relative to ECAP are still needed due to the lack of previous works. Moreover, no previous works were concerned with using ECMAP more than one pass. The current work aims to reach the following objectives.

First, predict the behavior of the load-displacement curves and peak load during the ECMAP and ECAP of Al-1050 using FEM. Second, investigate the effect of the ECMAP and ECAP on the strain distribution and deformation homogeneity. Third, compare the use of ECAP and ECMAP through analysis of the FEM results and verify them experimentally through the microhardness measurement, as hardness consider the most relevant property to the strain. Therefore, the main subject of the current work is the comparison between the using ECMAP or ECAP as SPD techniques in the processing of Al strips under the same imposed strain based on FEM simulations and verifying that experimentally. So, the current study will be the base for future work based on the microstructure and mechanical properties (tensile, compression, and impact) of ECMAP or ECAP samples.

## Materials and methods, and FEM simulations

**Fig. 1:**
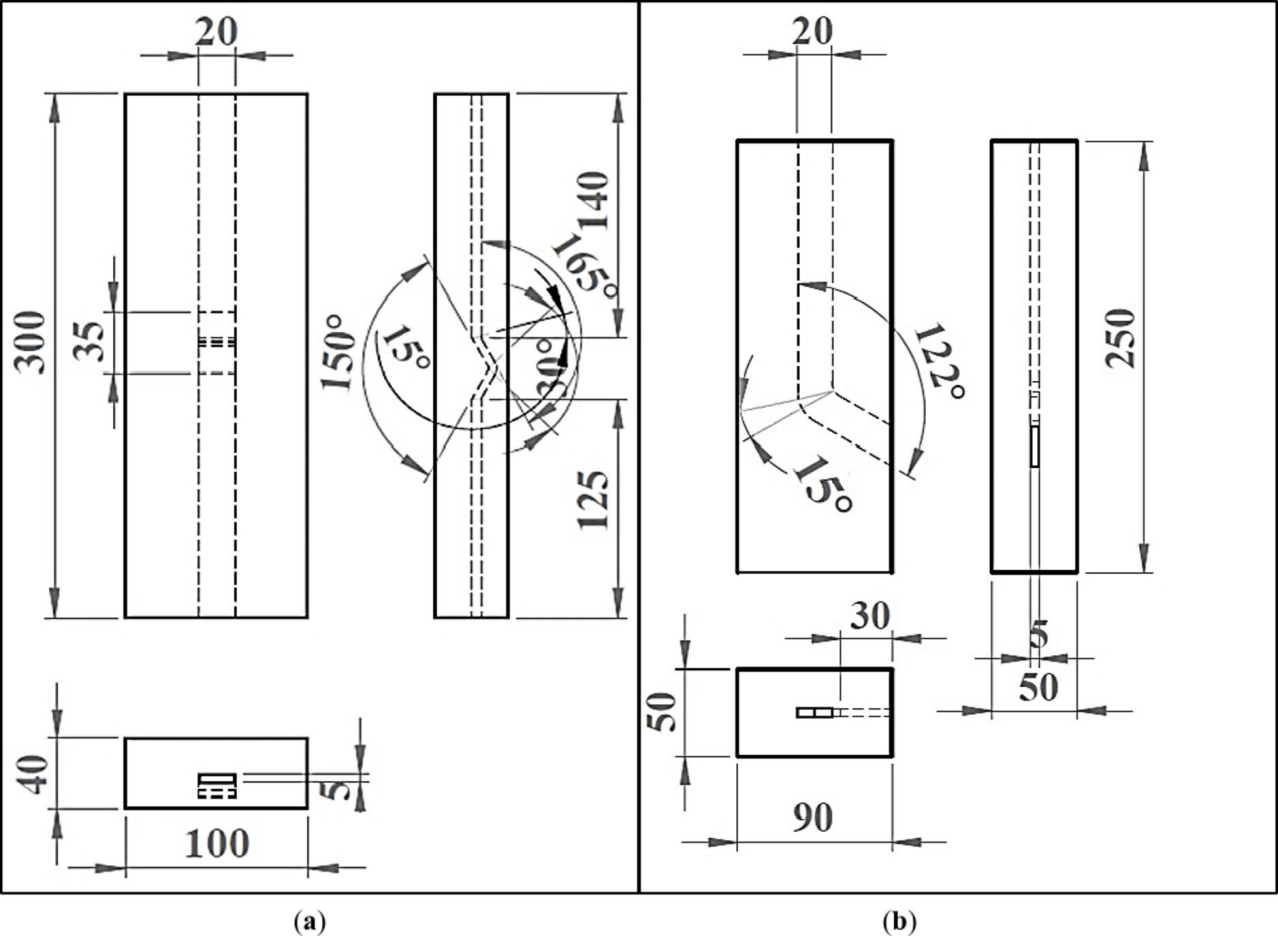
Drawing of (**a**) ECMAP and (**b**) ECAP dies (unit: mm).

Al-1050, with the chemical composition shown in Table 1, was used. The Al-1050 as-received samples were annealed and then machined using a wire cut machine to produce sheet samples with dimensions of 5 × 20 × 130 mm^3^ for the ECMAP and ECAP processing. The ECMAP and ECAP processes were carried out using split dies, as shown in Fig. 1. The ECMAP process of the Al-1050 was performed in the 3 ECAP stages. The ECMAP first and third stages are performed through inner die angle Φ = 165° and outer die angle ψ = 15°. The second or middle stage is performed through angles Φ and ψ = 150° and 30° (Fig. 1a). Therefore, the imposed strain in the first and third stages was equal to 0.151, and in the second stage is 0.301 according to the IWAHASHI Y Eq. (1)^14^. The approximate total strain imposed through the ECMAP die is 0.61. The ECMAP tail part, which was undeformed, was restored to continue the processing. The effective length used in the case of the ECMAP samples was 85 mm in length.

**Table 1:** Chemical composition of Al-1050 (wt%).

Element	Al	Si	Fe	Cu	Zn	Mn	Mg	Ti	V
Wt. %	99.76	0.037	0.09	0.051	0.018	0.019	0.012	0.005	0.008

1 $${\varepsilon _{ECAP~}}=N\left[ {\frac{{2\cot \left( {\frac{\Phi }{2}+\frac{\psi }{2}} \right)+\psi \cos ec\left( {\frac{\Phi }{2}+\frac{\psi }{2}} \right)}}{{\sqrt 3 }}} \right]~~$$

Where N, Φ and ψ are the number of passes, inner, and outer die angles, respectively.

On the other hand, a spilt die was used, with a channel of 5 × 20 mm^2^ cross-section with Φ = 122° and ψ = 15° in the ECAP process (Fig. 1b). The die construction imposed a strain of 0.61 on the sample per EACP pass according to the IWAHASHI Y Eq. (1)^15^. Both the ECMAP and ECAP processing were repeated up to 3 passes. The ECMAP and ECAP processes used molybdenum disulfide spray for lubrication and performed using 200 tons hydraulic press under a pressing speed of 1 mm/s at room temperature (RT). The load-displacement during the ECMAP and ECAP processing for the different number of passes was recorded, and the peak load in each pass was determined.

**Fig. 2:**
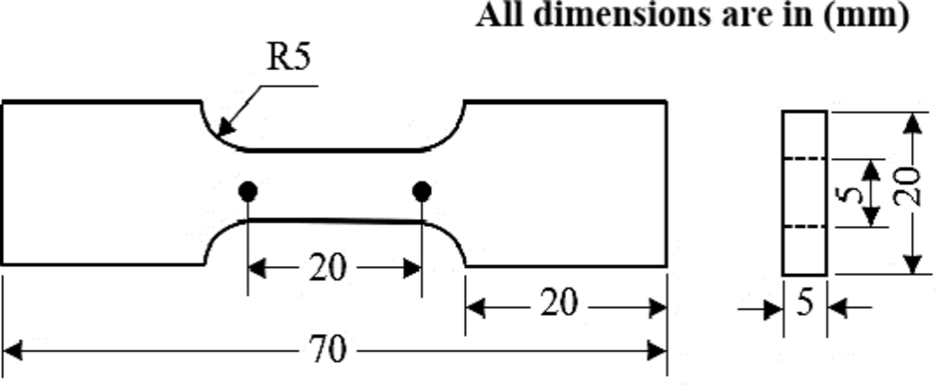
Drawing of the tensile test specimen (ASTM E8).

**Fig. 3:**
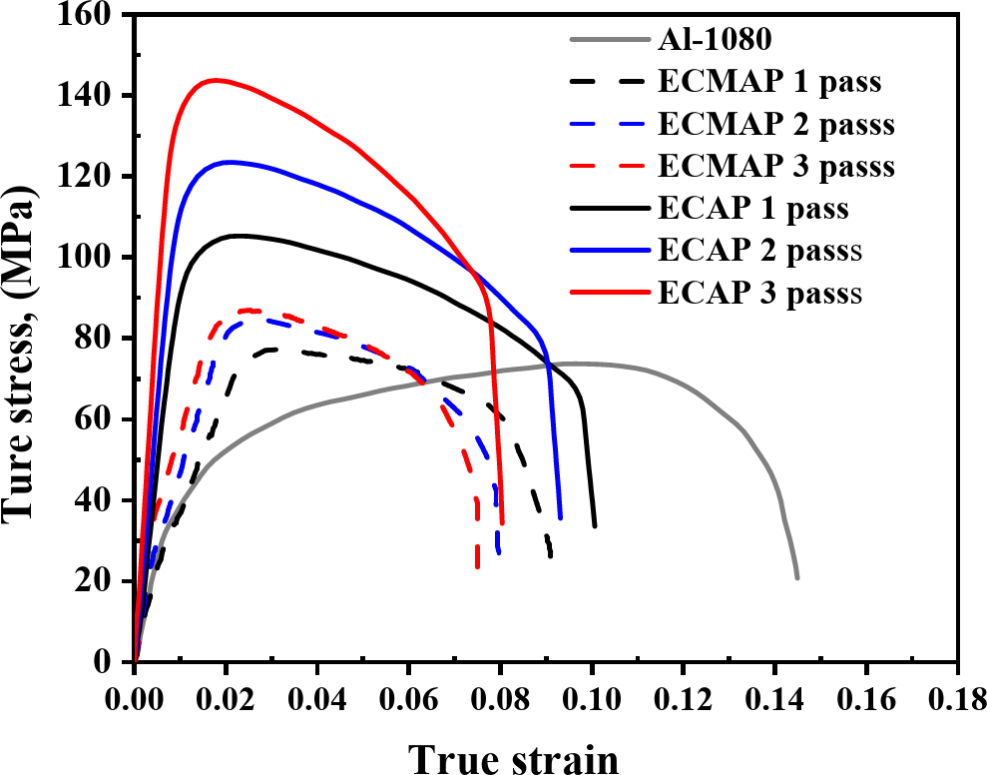
Tensile true stress-true strain curve of the different samples.

The Al-1050, the ECMAPed, and ECAPed samples were machined using wire cut to extract dog bone tensile samples with dimensions shown in Fig. 2, according to (ASTM E8). The tensile curves shown in Fig. 3 are used to upload the properties of the material needed for the FEM simulation. The tensile test was performed using a universal testing machine Instron model (4208-002) under a strain rate of 3 × 10^− 3^ s^− 1^.

**Fig. 4:**
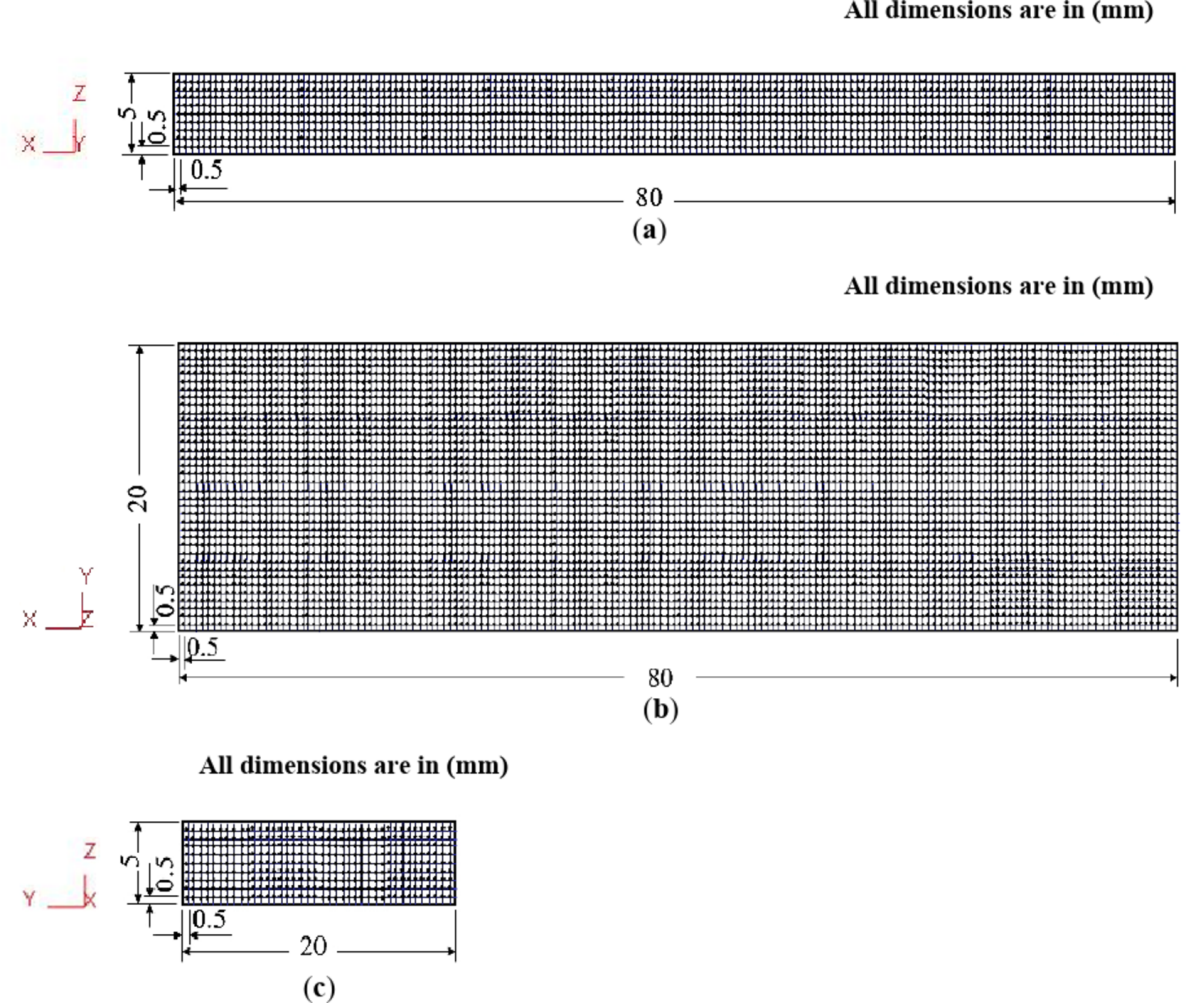
Microhardness measurement position in (**a**) ZX, (**b**) YX and (**c**) ZY plans.

The Al-1050, the ECMAPed, and ECAPed samples were sectioned along the pressing direction and through the width and thickness middle plans and so-called ZX, YX, and ZY plans for microhardness measurement. The microhardness samples were ground and polished up to shiny surfaces. The microhardness measurements were acquired across a network with a spacing of 0.5 mm for every two measurements, as shown in Fig. 4. The measured values were then drawn as color-coded maps. The Vickers microhardness was measured by a Mitutoyo microhardness tester under an applied load of 100 gf and a dwell time of 15 s. The average microhardness values are calculated based on the average of whole microhardness measurements in each plan. The deformation inhomogeneity index was obtained by calculating the standard deviation of the microhardness values. More details about the microhardness measurement and sample preparation can be found in previous works^2,3,6^. The tail part in the ECMAP is not deformed completely through passing in the whole deformation pass. So, the measurement of the microhardness in the case of the ECMAP and ECAP in the ZX, YX, and ZY plans is represented in areas, as shown in Fig. 4, with values of 5 × 80, 20 × 80, and 5 × 20 mm^2^.

FEM simulations were performed to obtain the load-displacement and effective strain distribution during the ECMAP and ECAP using the DEFORM-3D V.10.0^15^. The samples, dies, and punches were drawn and uploaded to the DEFORM-3D. The samples, dies, and punches were considered rigid plastic and rigid bodies, respectively. The material properties for each pass were extracted from the tensile curves shown in Fig. 3 and uploaded to the material library in the simulations program. The ECMAP and ECAP simulations were performed up to 3 passes. The data of each upcoming pass was interpolated from the last step in the previous one by using the batch-type method^2,16,17^. The samples meshed into 200,000 four-node elements to obtain highly reliable results.

The tolerance between the die, punch, and sample was automatically detected and revealed the interference depth between the punch and sample and limited to 0.7 mm. The ECMAP and ECAP sample top nodes received the pressing in the direction of the punch movement after the tolerance between them was automatically detected. A speed of 1 mm/s was applied for all passes. All the simulation processes were performed under a friction coefficient (COF) of 0.12 and RT. Automatic remeshing was activated when the elements became too distorted^2,16,17^.

The load-displacement curves and peak load values in each case were determined to be compared with the experimental one. Moreover, the strain distribution was obtained in the middle of the ZX, YX, and ZY plans. The calculations of the average effective strain of the ECAP samples in the middle of the ZX, YX, and ZY plans along the whole samples. In the ECMAP samples, the average values of the effective strain were obtained through areas of the sizes 5 × 80, 20 × 80, and 5 × 20 mm^2^ in ZX, YX, and ZY plans. The deformation inhomogeneity index was obtained by calculating the standard deviation of the strain values.

## Results and discussion

### Load-displacement

**Fig. 5:**
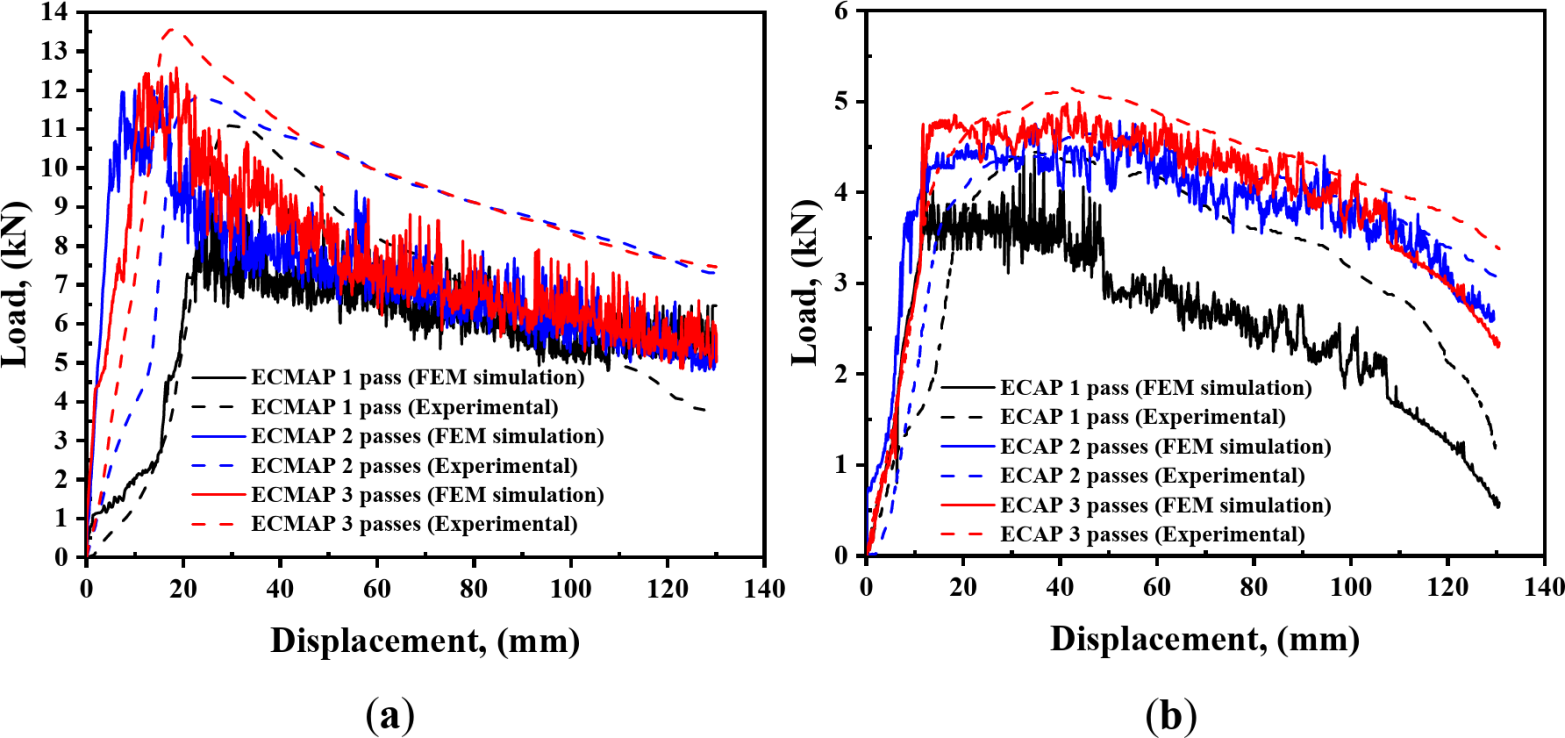
Experimental and FEM load-displacement curves of the samples deformed by (**a**) ECMAP and (**b**) ECAP.

Figure 5 shows the experimental and FEM load-displacement curves of both ECMAP and ECAP. In the case of the ECMAP, as shown in Fig. 5a, the load initially increased sharply after entering the first stage to around 1 kN. Then, the load increases at a lower rate or even at approximately steady stat around 2 kN. Then, the load sharply increases after punch pressing displacement reaches 17.5 mm, the sample enters the second stage, and the load value becomes 7.5 kN. Again, the load increases with a lower rate, and punch displacement reaches 30–35 mm. Finally, the load reaches its peak at a value of 11.1 kN. Then, the load starts to decrease with an oscillation in the load value until the punch finishes the total stroke displacement of 130 mm.

**Fig. 6:**
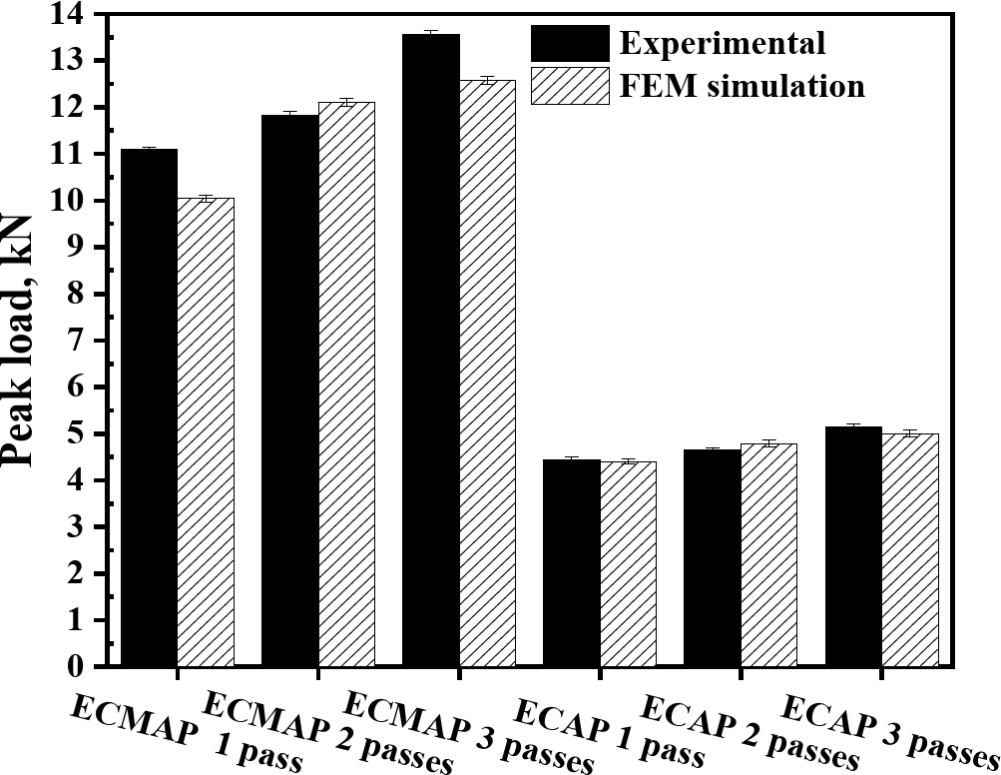
Experimental and FEM peak load of the ECMAP and ECAP samples.

The load behavior of the ECMAP samples is still the same with increasing the number of passes. However, the values of the displacement at which the load rate decreases or becomes a steady state case were shifted to the left direction of the curve, as shown in Fig. 5a. Moreover, the load value increases with the number of passes because of the strain hardening and dislocation density increase, as previously note during the SPD^18^. The peak load of the ECMAP samples of the FEM curves increases from 10.1 kN after one pass to 12.1 and 12.6 kN after 2 and 3 passes, as shown in Fig. 6.

The behavior of the ECMAP FEM and experimental load-displacement curves were so close to each other’s, as previously noted^12,13^. The FEM curves can introduce a more accurate representation of the load-displacement behavior considering the more data obtained through the FEM one. Experimental peak load values of ECMAP samples after 1, 2, and 3 passes of 11.1, 11.8, and 13.6 kN have a deviation ranging from 2.3 to 9.5% from the FEM, as shown in Fig. 6. Therefore, it can be noted that the FEM load curves results of the ECMAP indicate a high accuracy.

The load-displacement of the ECMAP curves was similar to those noted in previous works of ECMAP^10–13,18^. The load-displacement of ECMAP of Cu processed by two routes for two passes^10,11^, Al5754 processed through 3 stages^12,13^, and Al-1070 for two stages^18^ have the same behavior noted in the current study. However, a difference in peak loads and steady-state areas is due to the difference in the die’s geometry. Relative to the previous work of the comparison between the experimental and FEM of ECMAP^12,13^, the present work provided a lower degree of deviation. It can note that the deviation between the FEM and experimental peak loads of the ECMAP of Al5754 reached 13–21% in the first pass^12,13^, which is relatively higher than the deviation in the current study of 3-9.5% even after processing up to 3 passes. Interestingly, the FEM and experimental work of ECMAP (under the same condition materials, die, and processing conditions) indicate different peak loads^12,13^. Therefore, the 3D FEM of the ECMAP process in the present work can consider more accurate than the previous works^12,13^.

The load-displacement curves of the ECAP samples are shown in Fig. 5b. Both the experimental and FEM curves have the same behavior. Where the load increased sharply in the first 10 mm. Then, the load ramped and continued in a steady state mode for 40 mm. Finally, the load decreased gradually with a further increase in the displacement. The load-displacement curves in the current study have the same behavior noted during the ECAP of Cu, Al-1080, and Al-1050 after 1 pass^11,17,19–21^.

The behavior of the load-displacement curves after 2 and 3 ECAP passes was similar to that noted after the first pass^19^, with an increase in the load value, as shown in Fig. 6. The ECAP experimental peak load increased from 4.5 kN after 1 pass to 4.7 and 5.2 kN after 2 and 3 passes. A similar increase in the peak load is also noted in simulation results that match those of the previous results^11,19^. The peak load increase is due to the strain hardening and dislocation density increase^12,17,19^. Interestingly, the ECAP FEM peak loads deviated by 0.9–3.1% from the experiential one, which confirms the EFM results accuracy.

Through the comparison between the experimental and FEM load-displacement curves and peak loads of the ECMAP and ECAP, it can note the following. The peak load needed in the case of the ECMAP was higher by 149.4-163.4 and 127.9-151.8% in the case of the processing up to one to three passes relative to the ECAP process experimental and FEM results. Moreover, the flow of the load with the distance was smoother in the ECAP than in the ECMAP. Those observations can be explained by the complex path of the deformation that contains multiple stages in the ECMAP that hinder the material flow, and so increase the material hardening, that consequently increases the load. Furthermore, the different stages of deformation produce a back pressure effect that decreases the deformation homogeneity and increases the required load^10^. Moreover, the multiple steps during the ECMAP contribute to the increase of the friction between the die and the sample relative to the smoother path in the case of the ECAP processing, which increases the required load^17^. Therefore, the processing of Al-1050 strips is recommended to be performed using ECAP over ECMAP due to the lower load needed in the ECAP performed under the same strain imposed by the ECMAP process.

## Effective plastic strain distribution and homogeneity

**Fig. 7:**
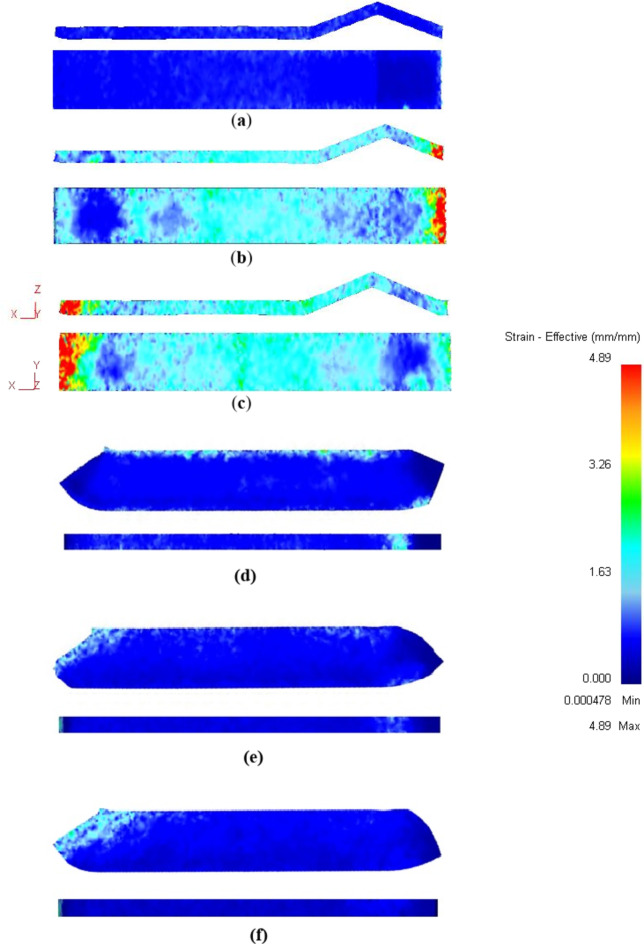
Effective plastic strain distribution contours in the ZX and YX plans of the ECMAP and ECAP samples deformed up to (**a** and **d**) 1 pass, (**b** and **e**) 2 passes, and (**c** and **f**) 3 passes.

**Fig. 8:**
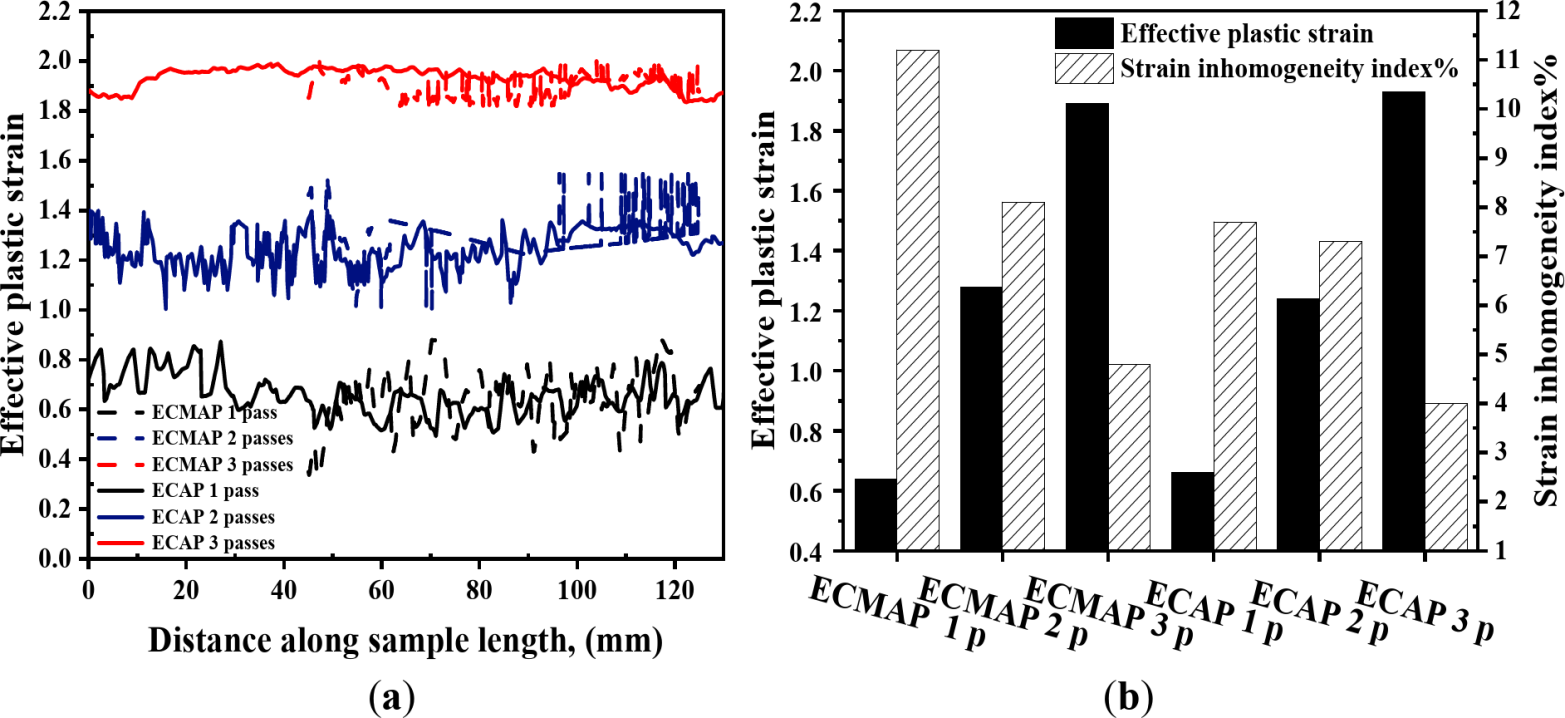
(**a**) Effective plastic strain distribution and (**b**) average effective stain values and strain inhomogeneity index of the ECMAP and ECAP samples in the YX plan.

**Fig. 9:**
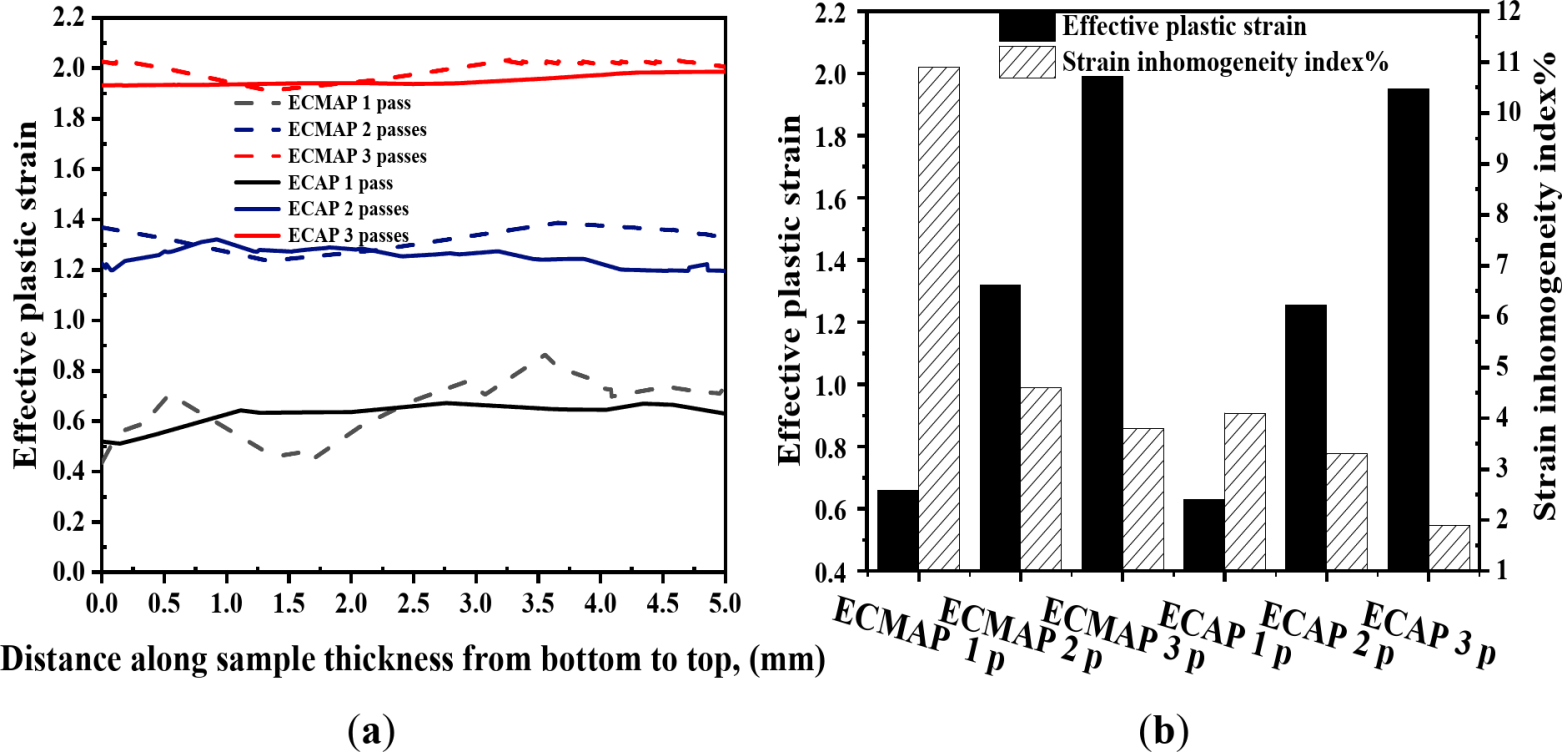
(**a**) Effective plastic strain distribution and (**b**) average effective stain values and strain inhomogeneity index of the ECMAP and ECAP samples in the ZX plan.

Strain distribution maps in the YX and ZX plans of the ECMAP samples are shown in Fig. 7. Strain distribution in both plans can be considered homogeneous. Strain values of the ECMAP samples were close to the strain obtained from Eq. (1) for each pass, as indicated by dash lines in Figs. 8a and 9a. Moreover, the average value along the YX and ZX plans of the ECMAP sample, as shown in Figs. 8b and 9b were close to that obtained through Eq. (1). The average strain obtained in the YX plan of 0.63, 1.27, and 1.89 deviated by 5 − 3% from the strain values of 0.61, 1.22, and 1.83 obtained from Eq. (1) after ECMAP from 1 to 3 passes, as shown in Fig. 8b. The average strain obtained in the ECMAP samples ZX plan of 0.66, 1.33, and 1.99 has a deviation of 7–9% from the strain values obtained from Eq. (1) after ECMAP from 1 to 3 passes, as shown in Fig. 9b.

More focus on the strain distribution in the YX and ZX plans noted in Figs. 8a and 9a indicate the following. The strain values were varied in the YX plan in the range of 0.88 − 0.33, 1.54–1.28, and 2.02–1.82, with differences in strain values of 0.55, 0.54, and 0.18 after 1, 2, and 3 passes. This observation indicates the decrease in the difference of the strain along the sample, proving the increase in the deformation homogeneity, as shown in Fig. 8b. The inhomogeneity index of the ECMAP in the YX plan decreased from 11.2, 9.2, and 4.9% after ECMAP up to 1–3 passes.

A similar observation of the decrease in the strain value variation in the ZX plan was also noted with an increase in the number of passes, as shown in Figs. 7 and 9. The strain was varied in the range of 0.86 − 0.44, 1.39–1.24, and 2.04–1.9, with strain differences of 0.43, 0.15, and 0.12 across the thickness after 1 to 3 passes. Furthermore, the strain inhomogeneity index in the thickness direction decreased from 10.9% after one pass to 4.4% after two passes and 3.8% after three passes, as noted in Fig. 9b. Interestingly, the homogeneity in the ZX plan was higher than the YX one due to the exposer to a high degree of deformation.

**Fig. 10:**
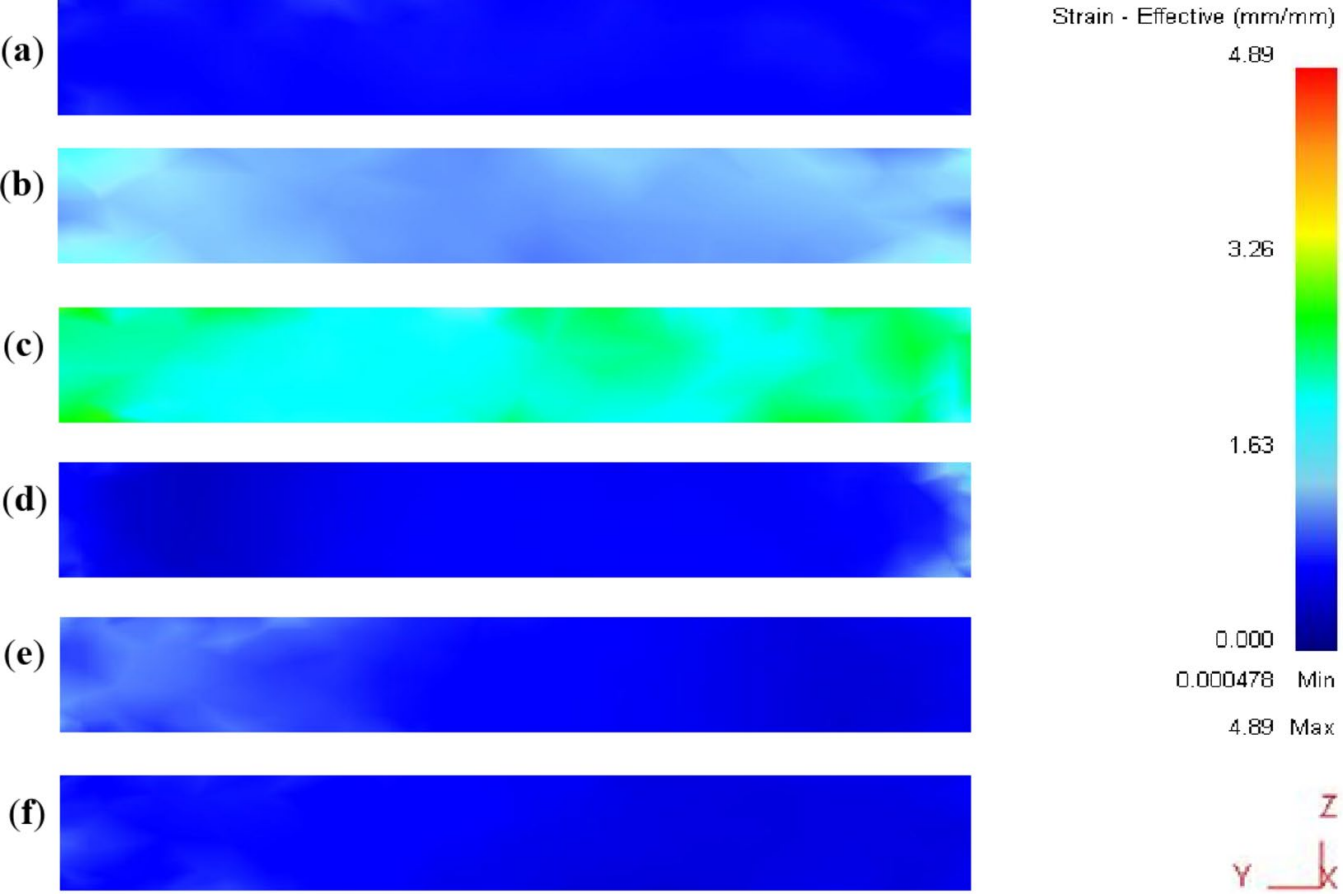
Effective plastic strain distribution contours in the ZY plan of the ECMAP from 1-3 passes (**a**,**c**) and ECAP from 1-3 passes (**d**,**f**) samples.

**Fig. 11:**
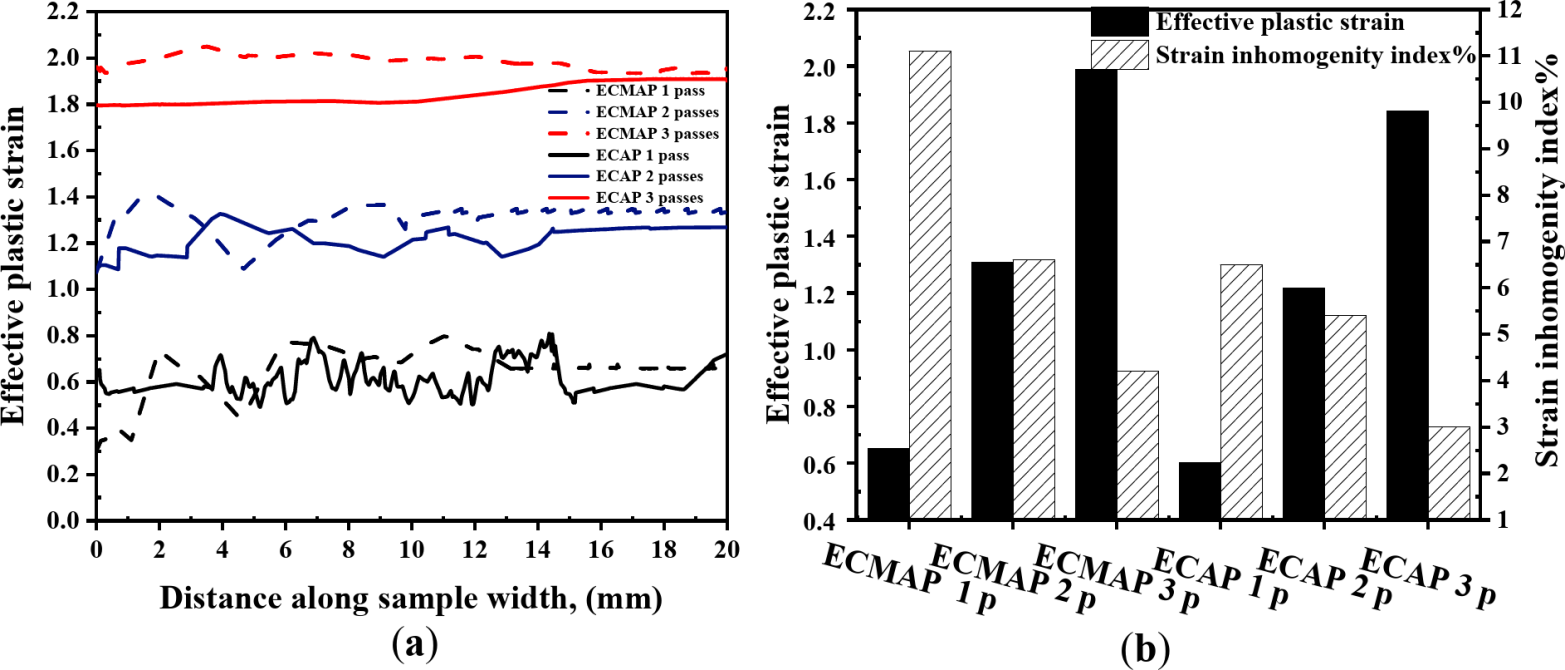
(**a**) Effective plastic strain distribution and (**b**) average effective stain values and strain inhomogeneity index along the ECMAP and ECAP samples in ZY plan.

The strain distribution of ECMAP samples in the ZY plan can considered homogenized, as shown in Fig. 10a-c. The strain values were varied through the range of 0.79 − 0.30, 1.4–1.07, and 2.05–1.93 after 1, 2, and 3 passes, as noted in Fig. 11a. The average strain in the ZY plan of 0.65, 1.31, and 1.99 after ECMAP up to 1–3 passes has a deviation of 7.1–8.5% from that of the Eq. (1). Furthermore, the strain inhomogeneity index decreased from 11.1% after one pass to 4.2% after three passes, as noted in Fig. 11b. Therefore, the ECMAP samples approximately homogenized in the three directions, with a close range of the strain inhomogeneity index in the three plans.

Relative to the previous works of the ECMAP processing of thin thickness Cu and Al alloy samples through 90° angle die^10,11^ and die with the same geometry of current work^12,13^, it can note the following. The FEM of ECMAP of Cu samples with thicknesses 6 and 10 mm shows the effectiveness of using route C^10,11^. Route C can provide a higher degree of homogeneity. The inhomogeneity index (standard deviation) of route C of 10% was lower than that of route A of 19%^10^. Interestingly, after modifying the route A die^11^, the inhomogeneity index value is still higher than route C. Therefore, route C in the current study contributes to obtaining a high degree of homogeneity (that is why it was selected in the present work). This observation was further proved through ECMAP of Al5754 with a thickness of 3 mm^12,13^. The standard deviation value of strain in the case of route A was 7.2%, which is higher than that of route C at 6%^12,13^. The previous results are consistent with the present work. However, the current work provides further information about strain distribution and deformation homogeneity for different ECMAP numbers of passes. Moreover, the average value of strain in previous works^10–13^ has a deviation of 17–25 and 11.5% from Eq. (1), which is higher than the maximum deviation of 9% in all plans in the current study, which confirms the accuracy of the FEM simulation results. This observation can be explained by the use of a high number of FEM elements through 3D simulations that can increase the accuracy. Considering that the accuracy of the FEM simulation improved through 3D FEM and the use of a fine mesh through increasing the number of elements.

The strain distribution maps of the ECAPed in the different plans are shown in Figs. 7d-f, 8a, 9a, 10d-f and 11a. The strain distribution of the ECAPed samples was more homogenized than that in the ECMAP one. More than 95% of the sample area has a strain value close to the average value in each plan. The strain values across the YX, ZX, and ZY plans were close to the average value across each plan of 0.66, 0.63, and 0.61 after one pass, as shown in Figs. 8b and 9b, and 11b, respectively. Moreover, the range of the strain variation across the ECAP sample’s different plans was smaller than that noted in the case of ECMAP samples. The average strain values in the different plans of the ECAP sample processed up to 1 pass have a deviation of 0.22–8.4% from that of 0.61 obtained from Eq. (1). The same observation was also noted after 2 and 3 passes, as the deviation of the average strains in the three plans were in the ranges of 0.22–2.8 and 0.6–6.6%. Therefore, like the ECMAP FEM results, the ECAP was performed with high accuracy. Similar to that noted in the ECMAP samples, the deformation homogeneity of the ECAP samples increased with the number of passes. An increase in the ECAP number of passes decreases the inhomogeneity index from 7.7 to 4%, 4.1–1.9%, and 6.5-3% after deformation from 1 to 3 passes in the YX, ZX, and ZY plans. Interestingly, similar to that noted in the ECMAP, a higher degree of homogeneity was observed through the ECAPed sample’s thickness relative to that in the YX and ZY plans and previously^20^. The inhomogeneity indexes of the ECAPed Al-1050 samples with thickness of 5 mm and length of 35 mm were 6 and 15% in the thickness and longitudinal directions^20^.

The ECAP results of Al-1050 agree with those of previous works on the ECAP of thin Al-1050 samples^19,20,22^. The ECAP of Al-1050 samples processed through die angles over 90° indicated strain inhomogeneity index (standard deviation of strain values) values lower than that of using a 90° die angle. The inhomogeneity index in the thickness direction in the present work was 4.1, 3.1, and 1.9% after the ECAP processing of Al-1050 samples with 3 mm thickness from 1 to 3 passes. However, the ECAP processing of Al-1050 samples with 5 and 2 mm thicknesses indicates an inhomogeneity index of over 90%^19^, 6%^20^, and 18%^22^ after processing to 3 and 1 passes. So, using a smaller ECAP angle provides a higher degree of homogeneity. This observation was also confirmed in previous work of the ECAP of Al-1050 samples with 2 mm thickness through die angles of 90^o^ and 120°^22^. The processing using angles of 90^o^ and 120° produces inhomogeneity indexes of 18 and 13%, respectively^22^.

The comparison between the strain distribution of the ECMAP and ECAP is in Figs. 8a and 9a, and 11a indicate the following. The variation or oscillation amplitude of the strain values around the average value for the ECAP samples was smaller than that of the ECMAP one. The strain variation range of the ECAP samples was smaller than that of the ECMAP one by 22.4–31.2, 17.2–62.4, and 1.5–35.7% for 1–3 passes in the YX, ZX, and ZY plans, respectively. This observation supports the strain inhomogeneity shown in Figs. 8b and 9b, and 11b for the different plans. The inhomogeneity index of the strain of the ECAP samples was smaller than that noted for the ECMAP by 19.4–30.8, 28.2–62.6, and 19-41.7% after 1–3 passes in the YX, ZX, and ZY plans, respectively. This observation can be explained by the longer path of deformation in the ECMAP, which increases the possibility of occurrence of grain recovery than that in the case of ECAP, which imposes an intensive strain. Moreover, the back pressure in the ECMAP die decreases the deformation homogeneity^10,11^. Where the back pressure contributes to the hindering of the material flow in the case of the ECMAP relative to the smoother flow noted in the case of the ECAP process. Therefore, the ECAP can be considered more effective in producing thin Al-1050 samples with a higher degree of deformation homogeneity than ECMAP. Moreover, the smaller range of the strain variation noted in the case of the ECAP samples relative to the ECMAP one in the different plans also contributes to the higher homogeneity in the case of the ECAP sample. The higher difference in strain values in the case of ECMAP and so lower homogeneity is due to using a larger value of Φ angles of the ECMAP. Therefore, increasing the number of passes through a larger channel angle die will not produce the same strain distribution as those obtained from a smaller angle die, as previously proven through FEM simulations^23^.

## Microhardness distribution and homogeneity

Hardness can be considered the most relevant mechanical property that can be integrated with the imposed strain of the deformed metals. The correlation between the strain and the hardness of SPD Al and Al alloys was investigated^2,16,17^. Moreover, the microhardness values of the cold deformed and the SPD materials were predicted with high accuracy based on the values of average imposed strain^24–26^. Therefore, the microhardness distribution, average value, and inhomogeneity index were obtained to confirm the effective strain results.

**Fig. 12:**
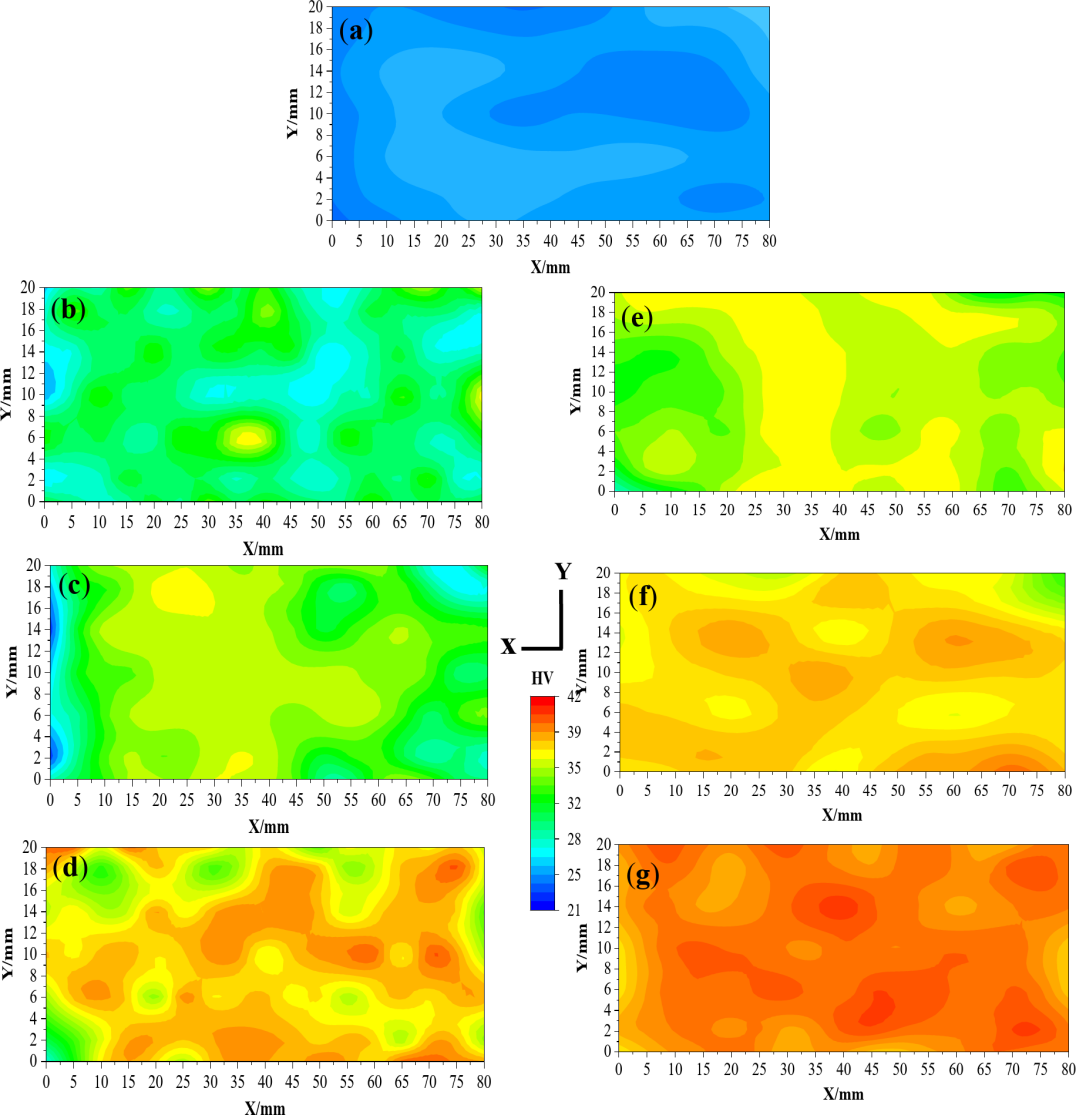
Microhardness distribution map in the YX plan of (**a**) Al-1050, ECMAP and ECAP samples deformed up to (**b** and **e**) 1 pass, (**c** and **f**) 2 passes, and (**d** and **g**) 3 passes.

**Fig. 13:**
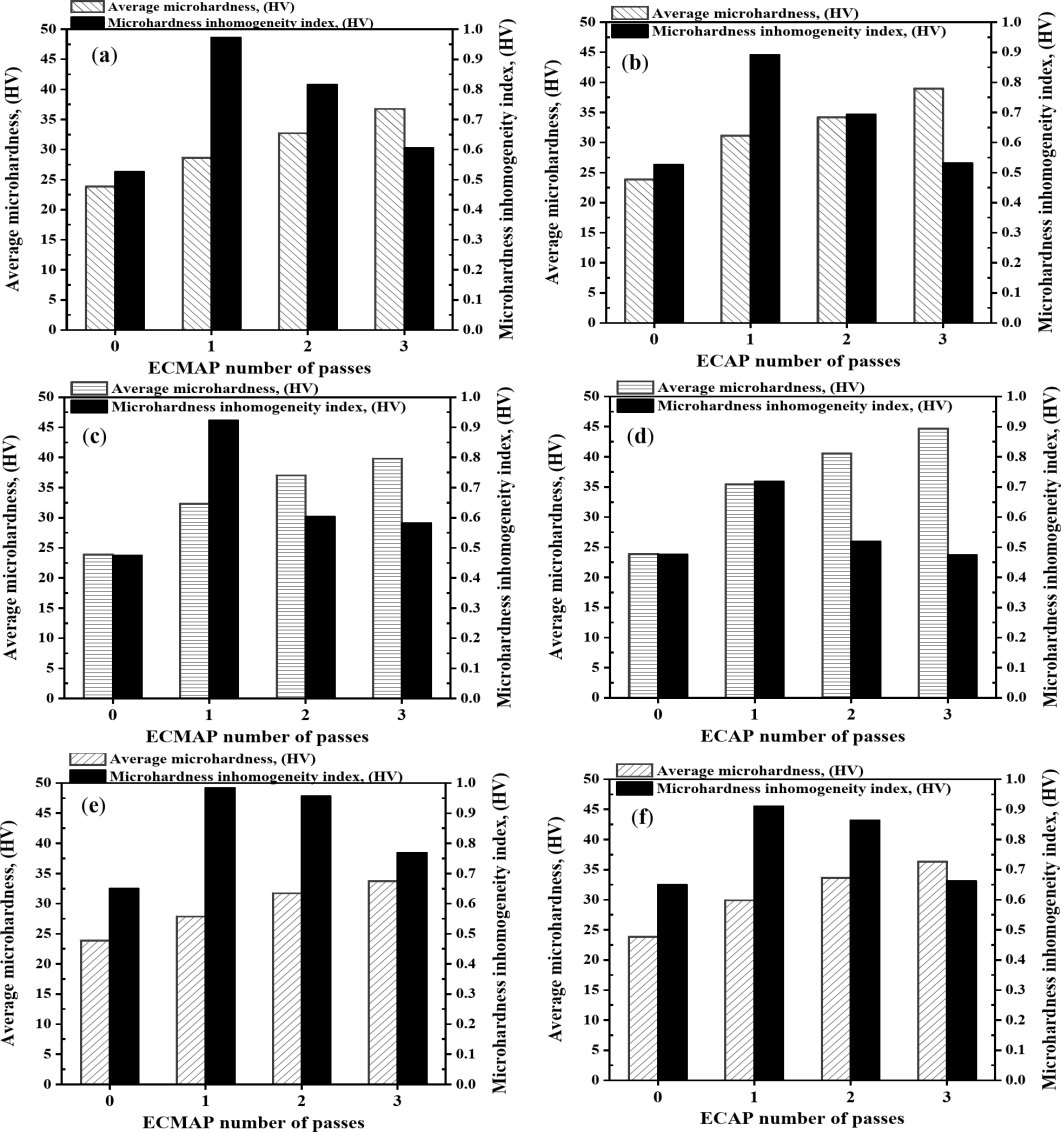
Average microhardness and microhardness inhomogeneity index of the ECMAP and ECAP samples in (**a**,**b**) YX, (**c**,**d**) ZX and (e-f) ZY plans.

Figure 12 shows the microhardness distribution maps in the YX plan for the Al-1050 and ECMAP samples. The microhardness distribution of the Al-1050 can be considered approximately homogenized. Over 70% of the sample area has a microhardness value close to the average value of the sample of 23.85 HV, as shown in Fig. 13a and b. Moreover, the average microhardness was close to those of maximum and minimum microhardness values of 26.1 and 22.42 HV, as noted in Fig. 12a. This observation can also be confirmed through the value of the microhardness inhomogeneity index shown in Fig. 13a-b which equals 0.53 HV. The microhardness distribution indicates a decrease in the homogeneity with a wide range of microhardness values after the ECMAP processing, as noted in Fig. 12b. The microhardness values increase to 28.7 and an inhomogeneity index of 0.97 after the ECMAP first pass, as indicated in Fig. 13a. Then, with an increase in the number of passes, the microhardness distribution homogeneity increases again. The area covered with the microhardness value close to the average value increased, as noted in Fig. 12b-d. The microhardness was increased by 20.1%, then by 14.2%, and finally by 12.4% after 1, 2, and 3 ECMAP passes, as shown in Fig. 13a. Therefore, the microhardness increased at a higher rate, then decreased with increasing the number of passes, with a decrease in inhomogeneity from 0.97 to 0.82, then to 0.61 HV after the ECMAP to 1, 2, and 3 passes.

The microhardness distribution of the ECAPed samples in the YX plan is shown in Fig. 12e-g. The ECAP samples have a relatively higher degree of homogeneity than the ECMAP samples. Around 62, 70, and 80% of the ECAP samples area have microhardness values near the average, as shown in Fig. 12e-g. The ECAP sample’s average microhardness after 1, 2, and 3 passes was 33.35, 36, and 40.94 HV, respectively, with an increase in microhardness by 30.7, 10, and 13.98%, as shown in Fig. 13b, with the same behavior noted with increase the ECMAP number of passes. The microhardness average values in the YX plan of the ECAP were higher by 4.5–8.8% than that of the ECMAP samples, as shown in Fig. 13a-b. Furthermore, the inhomogeneity index of the ECAP samples was lower by 8.1–15.2% relative to ECMAP, as shown in Fig. 13a-b. Therefore, the microhardness results confirm the FEM of the higher deformation homogeneity of the ECAP samples due to the intensive imposed strain during the ECAP. The gradual application of the imposed strain in ECMAP during processing improves the probability of the grain growth of the Al samples. Therefore, the possibility of recovery and increase in the grain size in ECMAP samples decreases their microhardness. Furthermore, the occurrence of recovery also contributes to the difference in the grain size, which consequently affects microhardness values and distribution across the sample and increases the inhomogeneity index. This explanation is consistent with that previously noted about using intensive strain in one pass and small angle Φ is more effective than imposing the same strain through multiple passes using a larger Φ angle^26,27^ .Where, achieving a large plastic strain in a sample by pressing several times through a die having a large value of Φ, is generally incapable of producing an array of ultrafine grains that may be achieved most readily by imposing a very intense plastic strain on the in each separate passage. Therefore, highly intensive strains can produce smaller grain sizes, so high mechanical properties (hardness) and deformation homogeneity are acquired.

**Fig. 14:**
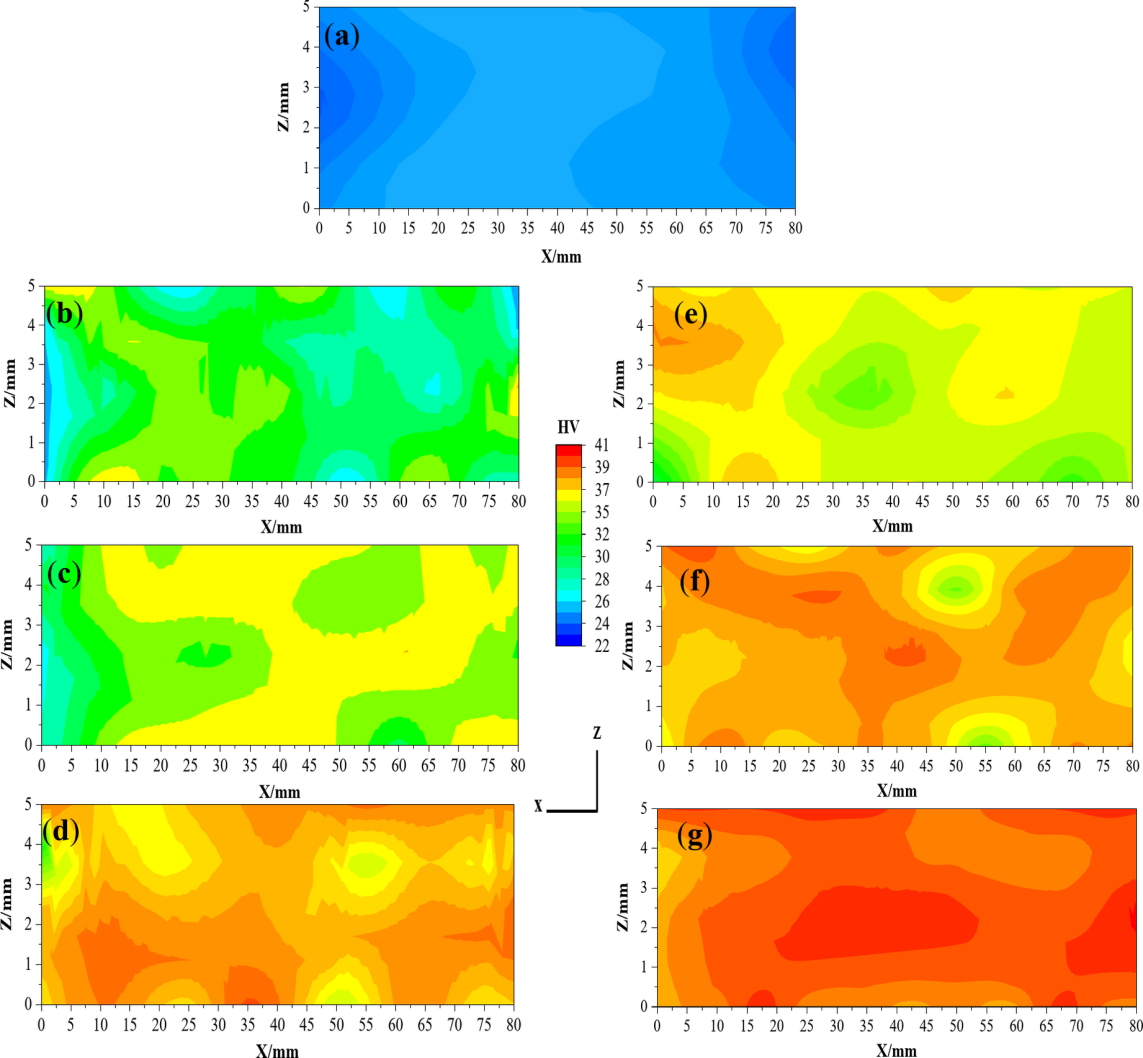
Microhardness distribution map in the ZX plan of (**a**) Al-1050, ECMAP and ECAP samples deformed up to (**b** and **e**) 1 pass, (**c** and **f**) 2 passes, and (**d** and **g**) 3 passes.

The microhardness distribution in the ZX plan is shown in Fig. 14. The microhardness and deformation homogeneity were also increased with the number of passes in the ZX plan. The microhardness of the ECMAP samples was increased by 35.5, 14.5, and 7.5% after 1, 2, and 3 passes, as shown in Fig. 13c. On the other hand, the ECAP contributes to increasing the microhardness by 48.3, 14.5, and 10.2% after ECAP up to 1, 2, and 3 passes, as observed in Fig. 13d. Therefore, the microhardness of the ECAP samples is higher by 9.5–12.3% over that of the ECMAP samples in the ZX plan. Interestingly, the deformation homogeneity of the ECAP samples was also higher by 13.9–22.2% than that of the ECMAP one, as shown in Fig. 13c, d, and 14. Therefore, the deformation homogeneity of the ECAP was higher than that of ECMAP, which agrees with the effective strain results in the ZX plan.

**Fig. 15:**
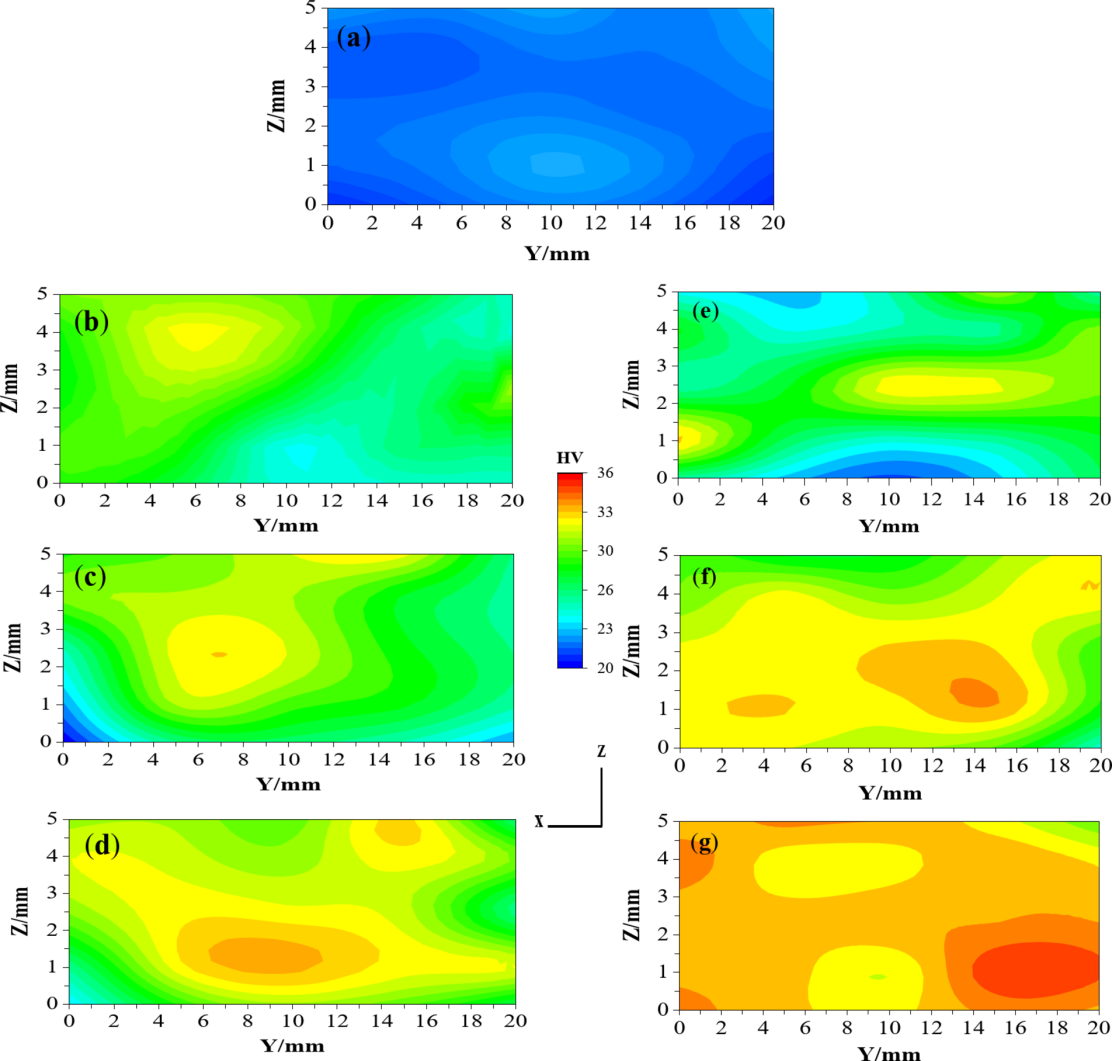
Microhardness distribution map in the ZY plan of (**a**) Al-1050, ECMAP and ECAP samples deformed up to (**b** and **e**) 1 pass, (**c** and **f**) 2 passes, and (**d** and **g**) 3 passes.

Interestingly, the previous works of the ECMAP and ECAP were concerned with the microhardness measurement in the ZY plan (perpendicular to the pressing load). The microhardness distribution homogeneity in the ZY plan was increased with increasing the number of passes with a higher degree of homogeneity of the ECAP one, as noted in Fig. 15. The homogeneity of the ECMAP was increased with increasing the number of passes, as shown in Fig. 15. The inhomogeneity index of the ECMAP decreases from 0.99 HV to 0.96 HV and then to 0.77 HV after 1, 2, and 3 passes, as shown in Fig. 13e. The microhardness inhomogeneity index of the ECAP samples was lower by 7.5–14% relative to that of the ECMAP, as shown in Fig. 13e, f. The present results also confirm the strain inhomogeneity results, especially in the ZY plan.

The microhardness average value of the ECMAP samples in the ZY plan was increased by 16.8%, then by 13.9%, and finally by 6.3% after processing up to 1, 2, and 3 passes, as shown in Fig. 15 and summarized in Fig. 13e. This behavior of high increase in microhardness followed by a lower rate of increase was also observed in the ECAP samples. The microhardness was increased by 25.5, 12.4, and 8.1% after the processing up to 1, 2, and 3 passes, as shown in Fig. 13f. Considering that the microhardness of the ECAP was higher by 6-7.5% over that of the ECMAP samples, as shown in Fig. 15. A similar observation of the high increase followed by a lower rate of increase was also noted after the ECMAP and ECAP of Al99.99%^9^. The microhardness increased by 52.1%, then by 11%, and finally decreased by 1% after ECMAP up to 1, 2 and 3 passes^9^. On the other hand, the microhardness was increased by 109%, then by 5.5%, and finally decreased by 1.9% after ECAP up to 1, 2, and 3 passes of Al 99.99%^9^. Those observations agreed with the effect of the saturation in the grain size (no further decrease in the grain size can obtained with the increase of the passes or the imposed strain, but directed to evolve into an equiaxed grain size structure) with the increase of the strain during the ECMAP and ECAP, as previously noted^9,28^. The increase of the microhardness can be related to the increase of the imposed strain with increasing the number of passes. Initially, grain refinement can be considered obvious, which leads to a decrease in grain size and an increase in the dislocation density that increases microhardness at a high rate. With increasing the number of passes, the grain size and dislocation density can reach saturation, so the increase in microhardness occurs at a low rate^9,25^. This observation was also confirmed during the ECAP of A199.99%, Al-1050, and Al-1070 using different processing routes^27–29^. The microhardness was increased by 117, 9.2, and 0.2% after ECAP to 1, 2, and 3 passes of Al 99.99%^28^. Moreover, the microhardness was increased by 125%, then by 23.3 and 22.2%, finally decreasing or even increasing by 1 and 1.5% after ECAP up to 1, 2, and 3 passes of Al-1050 using routes Bc and C, respectively^29^. This observation was also observed in ECAPed Al-1070 samples, as the microhardness was increased by 85.7%, then by 15.7%, and finally by 7.6% after ECAP up to 1, 2, and 3 passes^27^. The percent increase of the microhardness in the previous work was higher than that of the current study after the initial pass due to the higher ECAP die angle used in different previous works^9,27–29^.

Interestingly, the notice of the higher microhardness of the ECAP relative to the ECMAP by 4.5–8.8, 9.5–12.3, and 6-7.5% in the YX, ZX, and ZY plans was also observed in the Al99.99%^9^, that consider the only previous work compare between the ECMAP and ECAP processing. The microhardness of the ECAP samples was higher than that of ECMAP by 5.6–11.8% in the ZY plan^9^, which supports the current results of the effectiveness of the ECAP in improving the Al-1050 sample microhardness relative to using ECMAP. Furthermore, the increase in strain improves the deformation homogeneity of ECMAP and ECAP, as shown in Figs. 8b and 9b, and 11b.

The higher microhardness average values and homogeneity of ECAP over that of ECMAP samples can be explained by the complex path of the deformation that contains multiple stages in the ECMAP that hinder the material flow. The back pressure induced during multiple stages of the ECMAP decreases the deformation homogeneity. Moreover, the longer path of deformation during the ECMAP increases the possibility of the occurrence of grain recovery than in the case of ECAP. In the ECAP, an intensive strain is imposed that produces a smaller grain size and higher microhardness than the ECMAP samples^26^. The improvement in the homogeneity based on the microhardness (Fig. 13) in the different plans, with an increase in the number of ECAP passes, as previously noted through the cold forming and ECAPed Al-1080^16,17,25^, proves the results of the strain homogeneity. Therefore, the present results of the increase of the deformation homogeneity, with increasing the ECMAP and ECAP number of passes proved through the strain and microhardness homogeneity, can also prove as previously obtained in FEM and experimental works of different Al pure and Al samples^2,16,17,25,28^.

## Conclusions

The current study, that cover of the experimental and FEM simulations of ECMAP and ECAP of Al-1050 samples, can conclude the following.

- The ECMAP and ECAP processing of Al-1050 samples was performed successfully under the same imposed strain to 3 passes. However, the ECAP process provided easier and faster processing with a smoother material flow than the ECMAP.
- Due to the complex path of the ECMAP processing, the ECMAP experimental and FEM peak loads were higher by 149.4-163.4 and 127.9-151.8% than that of the ECAP, which confirms the easier processing using ECAP. FEM simulations predicted the load-displacement curves behavior with high accuracy and a deviation of 2.3–9.5 and 0.9–3.1% of the FEM peak loads values of the ECMAP and ECAP from the experimental values.
- FEM average effective plastic strain was obtained with high accuracy and a deviation in the range of 3–9 and 0.22–8.4% in the different plans for different passes of the ECMAP and ECAP samples relative to the IWAHASHI Y equation strain.
- The ECAP sample’s homogeneity in the form of strain was higher by19.4-30.8, 28.2–62.6, and 19-41.7% after the processing up to 1–3 passes in the YX, ZX, and ZY plans than that of the ECMAP samples, which confirmed by the microhardness homogeneity and so verified the FEM strain results.
- The ECAP processing of Al-1050 strips is more effective than the ECMAP in producing larger-size samples under a lower applied load with a higher degree of homogeneity and microhardness.

## Data Availability

The datasets used and/or analyzed during the current study available from the corresponding author on reasonable request.
