# Publisher Correction: Severe plastic deformation of Al strips through equal multi and single channel angular pressing

**DOI:** 10.1038/s41598-025-94141-8

**Published:** 2025-03-26

**Authors:** Elshafey Ahmed Gadallah, Mohamed Ibrahim Abd El Aal

**Affiliations:** 1 Mechanical Department (Production), Faculty of Technology and Education, Suez University, Suez, 43221 Egypt; 2 Department of Mechanical Engineering, College of Engineering in Wadi Alddawaser, Prince Sattam Bin Abdulaziz University, Wadi Addawaser, 18734 Saudi Arabia

Correction to: *Scientific Reports* 10.1038/s41598-025-88185-z, published online 15 February 2025

The original version of this Article contained an error in the spelling of the author Mohamed Ibrahim Abd El Aal.

In addition, Mohamed Ibrahim Abd El Aal was incorrectly affiliated with ‘Mechanical Department (Production), Faculty of Technology and Education, Suez University, Suez 43221, Egypt’. The correct affiliation is listed below.

Department of Mechanical Engineering, College of Engineering in Wadi Alddawaser, Prince Sattam Bin Abdulaziz University, Wadi Addawaser 18734, Saudi Arabia.

The original Article has been corrected.

